# Genome Sequence of Australian Indigenous Wine Yeast Torulaspora delbrueckii COFT1 Using Nanopore Sequencing

**DOI:** 10.1128/genomeA.00321-18

**Published:** 2018-04-26

**Authors:** Federico Tondini, Vladimir Jiranek, Paul R. Grbin, Cristobal A. Onetto

**Affiliations:** a Department of Wine & Food Science, University of Adelaide, Adelaide, South Australia, Australia; b Australian Research Council Industrial Transformation Training Centre for Innovative Wine Production, Adelaide, South Australia, Australia

## Abstract

Here, we report the first sequenced genome of an indigenous Australian wine isolate of Torulaspora delbrueckii using the Oxford Nanopore MinION and Illumina HiSeq sequencing platforms. The genome size is 9.4 Mb and contains 4,831 genes.

## GENOME ANNOUNCEMENT

Torulaspora delbrueckii occurs saprophytically on wine grape surfaces worldwide (1). Under winemaking conditions, it displays a less vigorous fermentation phenotype than Saccharomyces cerevisiae, differing in flavor and aroma compound production (2). Its favorable oenological traits, such as low acetic acid production and osmotic tolerance, have led to its commercialization and adoption for use in the wine industry (3). Despite this, T. delbrueckii is still not well characterized at a molecular level, with no other genomes reported from wine isolates.

For a better understanding of T. delbrueckii oenological traits, RNA sequencing (RNA-seq) studies under wine-like conditions are required. However, in the absence of a closely related reference sequence, significant challenges remain when it comes to assembling short reads into full-length gene and transcript models. In order to facilitate future wine-related studies, the genome of a wine isolate of T. delbrueckii was sequenced and characterized.

T. delbrueckii strain COFT1 was isolated from a spontaneous wine fermentation at the Yalumba Wine Company (Angaston, South Australia, Australia). High-molecular-weight genomic DNA was extracted according to the phenol-chloroform protocol (4), including a 2-h preincubation with Zymolase. DNA was prepared for sequencing with the MinION device using the SQK-LSK108 library prep kit (protocol GDE_9002_v108_revT_18Oct2016) and R9.4 chemistry. A total of 138,992 reads were obtained, for a total of 1,214 Mbp (130× coverage) and an average length of 8,737 bp. Fast5 files were base called using Albacore version 2.0.2. Passed reads were trimmed for adapters using PoreChop version 0.2.3 and then assembled using SMART*denovo* version 1.0 (https://github.com/ruanjue/smartdenovo). Contigs obtained from the assembly were polished using Racon version 0.5.0 (5) and Nanopolish version 0.8.5 (6). A final polish of the assembly was performed with Pilon version 1.22 using Illumina HiSeq RNA reads extracted from pure culture laboratory ferments with strain COFT1. The final assembly had no gaps, with a total length of 9,356,826 bp arranged in 9 chromosomes (1 mitochondrial chromosome) and an average GC content of 42%. The genome was first annotated with YGAP (7), and 5,231 genes were predicted. An improved annotation was performed with MAKER2 (8), providing STAR RNA alignment information (9) for the prediction of protein-coding genes. A total of 4,831 protein-coding genes were identified, compared to 4,714 and 4,972 protein-coding genes reported in previous genomes (10, 11). Functional annotation of the predicted protein sequences was performed using BLASTP (12) against the Swiss-Prot protein sequence database (E value = 1e^-5^) (13). BUSCO assessment (14) revealed a genome completeness of 98%.

The functional annotation of the genome, as well as its completeness, allows comparison with the species S. cerevisiae and a better understanding of the oenological traits of this yeast. The genome sequence reported here will assist in delivering clearer transcriptional results in complex wine-like fermentations (e.g., mixed fermentations), providing useful insight into these processes for the wine industry.

### Accession number(s).

The final genome sequence has been deposited to NCBI GenBank database under accession numbers CP027647 to CP027655.
